# Effects of Socioeconomic Status, Parental Stress, and Family Support on Children’s Physical and Emotional Health During the COVID-19 Pandemic

**DOI:** 10.1007/s10826-022-02339-5

**Published:** 2022-07-04

**Authors:** Sara Scrimin, Libera Ylenia Mastromatteo, Ani Hovnanyan, Benedetta Zagni, Enrico Rubaltelli, Tiziana Pozzoli

**Affiliations:** Department of Developmental and Social Psychology, University of Padova, Padua, Italy

**Keywords:** COVID-19, Socioeconomic status, Parental stress, Children’s physical and emotional health, Parental support

## Abstract

In the current study, we conduct an exploratory study on children’s emotional and physical health in response to the COVID-19 pandemic. The direct and interactive effects of parental stress, family socioeconomic status (SES), and family support on child adjustment were investigated. A total of 116 children of varied socioeconomic and their parents were interviewed. Parents with low household income perceived greater distress related to uncertainty and health worries compared to those with higher household income. However, it was among high-SES families that parental distress was associated with child difficulties. At a multivariate level, children’s health was associated with SES, family support, and parental COVID-19 stress. Among families with low household income, when parents perceived low/average COVID-19 stress, family support worked as a protective factor for children’s adjustment. Understanding how COVID-19 relates with children’s emotional and physical health within families with low and high household income may help to inform recommendations for best practices, for example through family support interventions.

## Highlights

- The pandemic represents a significant challenge to children and family well-being.
- Parental stress due to the pandemic and lockdown is linked with children physical and emotional discomfort.
- While dealing with Covid-19 low SES households are more at risk than high SES due to uncertainty and health worries.
- Children’s perceived family support is an important protective factor within low SES households.

Given the rapid global increase in the number of COVID-19 cases, on February 23, 2020, the Italian government was forced to close all schools and impose severe restrictive measures to limit transmission of the virus. From the beginning of March, all Italian families were forced into a mass lockdown. Parents and children soon had to deal with challenges related to the uncertainty and fear of the virus, as well as significant changes in family routines (i.e., they had to handle their children’s online lessons, work in atypical settings and take care of home chores). Children had to face the lack of classroom life and social face-to-face interactions while forced to remain within their homes, with restrictions in space and activities.

Recent studies have suggested that the pandemic has had important consequences on individuals’ mental health (Barari et al., 2020; Mazza et al., 2020; Moccia et al., 2020; Rossi et al., 2020). Data on the effects of the virus propagation and consequent restrictive measures on school-age children and their parents have underlined a number of difficulties related to psychological functioning (Brooks et al., 2020; Loades et al., 2020; Orgilés et al., 2020; Romero et al., 2020; Spinelli et al., 2020) and health problems (e.g., variation in sleep patterns, Cellini et al., 2021). In addition, it has been found that during the lockdown parents have been exposed to several sources of minor and major stressors (Fontanesi et al., 2020). Caregivers have been expected to care for kids 24/7 while attempting to manage the household, work remotely, and/or deal with the daunting chance of losing their job. Meanwhile, they have had no clarity on how long the situation will last and have had to deal with constant uncertainty related to the spread of the virus. For parents living in poor communities and low-income and crowded households, these challenges have likely been exacerbated (Van Lancker & Parolin, 2020).

Extensive literature documents how low socioeconomic status (SES) adversely affects family and children’s psychological functioning (Chaudry & Wimer, 2016; Kaiser et al., 2017; Peverill et al., 2021; Spencer et al., 2013). Following the American Psychological Association definition (American Psychological Association (2007)), we here consider high and low family SES as an economic and social position determined by the composite of their economic and social position in relation to others, based on total houseold income, parental education, and occupation (particularly in relation to unemployment rates of the parents). Living in poor households is a major risk factor for several mental, emotional, and behavioral disorders, as well as other developmental challenges and physical health problems (Pinderhughes et al., 2001). The family stress model shows how economic hardships and pressures exacerbate child maladjustment primarily through parents’ psychological distress, interparental relationship problems, and disrupted parenting (Masarik & Conger, 2017). More specifically, financial strain tends to undermine positive parental behaviors, such as warmth and consistent discipline, as well as to increase harsh interactions and scarce and inconsistent support within the family (Conger et al., 2010). Within these environments, prolonged confinement, fears of infection, frustration and boredom, lack of personal space at home, and the potential additional source of stress due to important financial loss caused by the pandemic can be even more problematic and have enduring effects on children.

Data on the consequences of pandemics show that most children have experienced psychological discomfort (Brazendale et al., 2017; Brooks et al., 2020; Jiao et al., 2020; Liu et al., 2020; Wang et al., 2020). Furthermore, evidence suggests that when children are out of school, they are physically less active and have much longer screen time, irregular sleep patterns, and less healthy diets (Sprang & Silman, 2013; Wang et al., 2019). Such negative effects on health are likely to be much worse when children are constricted to their homes without chances to play outside, interact with peers, or spend time within the school context. A wide range of studies assessing psychological functioning among the pediatric population have been published following the COVID-19 pandemic and related lockdown (Loades et al., 2020; Racine et al., 2020), however most of these works focus on pre-adolescents or adolescents (age range between 10 and 18) and relay on self-report measures (see Loades et al., 2020 for a review). Data on younger children’s psychological distress are scarce and report the presence of psychological difficulties in children during the COVID-19 pandemic, with fear, clinging, and irritability as the most severe symptoms for younger children, as assessed by parents (Jiao et al., 2020). Yet, up to now most, if not all studies, have used convenience sampling and online questionnaires (see Loades et al., 2020; Panchal et al., 2021 for a review). Such methodologies are not always easy for school-age children to understand particularly when collecting information from younger children that may have poor reading and comprehension skills. Although it is paramount to ask children to report on their own health to understand the trajectories of health and illness (Riley et al., 2004), it is also important to be sure that they comprehend the items and rating scales. Self-report measures can generally be used with children who are old enough to understand and use self-report scales, are not overly distressed, or cognitively impaired (Riley, 2004). Yet not all these requirements are met when faced with a pandemic and especially with a related lockdown, within very crowded and poor households. For this reason, interviews might allow for gathering more reliable data. Additionally, there is a limited validity of information provided by proxy-respondents (Jensen et al., 1999; Looker, 1989) that are often not equivalent to that reported by both children with chronic health conditions and healthy children (Yeh et al., 2005; Vance et al., 2001). This is particularly true when parents are under stress or dealing with a crisis in which case, they are likely to under or over report their child’s emotional problems (Stover et al., 2010).

Because of COVID-19, parents have experienced increased stress and fear, which might have challenged their capacity for tolerance and for perspective planning. This in turn might have exposed children to direct and indirect stressors such as fear and worry about the pandemic, feelings of severe uncertainty about the future, and hassles in handling everyday challenges in a changed and pressured family environment (Spinelli et al., 2020). Indeed, it is well known that family stress can impact child well-being (Dalton et al., 2020), which might be particularly true when facing a pandemic that is perceived by children mostly through adults (e.g., through the media, parental narratives, and parental limitations). Hence, cumulative exposure to stressors related to the spread of the virus, lockdown, and parental stress can negatively affect a child’s development and well- being (Prime et al., 2020), especially among families with low household income. As widely reported in the literature, more disadvantaged families that already carry the cumulative consequences of chronic sources of stress may be more vulnerable and have fewer protective factors also when confronted with the new challenges of a pandemic. As reported by Masten and Motti-Stefanidi (2020) in terms of COVID-19, there should be concern about children who were already struggling with the developmental threats associated with poverty and other secondary adversities. Yet, even if both children and their parents may be in a time of hardship, they might be able to handle better the daily sources of stress (Masten et al., 1999). That is, on the short-term children living in low-SES households could be able to respond more rapidly and to adjust to the changes and challenges by handling them in the same way they use to handle their previous sources of stress. However, on the long term the cumulative effect of multiple exposures could have greater consequences on children well-being.

Although it has been documented that stress exposure and parents’ difficulties are linked to children’s psychological problems (Cobham et al., 2016), it is also true that times of hardship can allow for creative opportunity, for example, to build stronger relationships with children or support their resilience. Moreover, most children who encounter adversities are capable of adapting and functioning adequately (Masten et al., 1999). The degree of adaptation in relation to the amount and severity of family stress and adversities is determined by the presence of important protective factors, such as supportive family environments (Holahan & Moos, 1985). Impoverished living conditions and low SES have been shown to negatively affect parenting quality, therefore representing important risk factors for children’s health (e.g., Bornstein & Bradley, 2014), whereas the presence of at least one caring parent who is a constant and supportive presence in the life of a child works as a strong protective factor (e.g., van Harmelen et al., 2016). A supportive family environment providing warmth, responsiveness, and nurturance is linked to a host of positive developmental outcomes, including socio-emotional competence, academic success, and good physical health (Newland, 2015). Prior research has shown that family support has positive effects on children (Schofield et al., 2012), and may reduce stress among youths (Chukwuorji et al., 2017). For example, a very recent work has shown how during COVID-19 lockdown in China parent-child open and supportive communication helped children and adolescents cope with mental health problems (Tang et al., 2021). Furthermore, based on a functional model of social support processes (Wills & Cleary, 1996), family support can help children to cope more with the life-challenges and can be an important aspect working to improve adjustment to various life stressors (Chukwuorji et al., 2017; Manczak et al., 2018). Even more importantly, the observed benefits of consistent family support and stimulation tend to be more pronounced for low‐income children (Crosnoe et al., 2010). Research has shown that in impoverished families, family support may be compromised due to the stressful situation resulting from financial strain, joblessness, and limited access to essential goods and opportunities (Bornstein & Bradley, 2014). Hence, when despite the socio-economic difficulties, parents are able to provide a responsive and supportive environment to their children this can play a significant stress-buffering effect (McConnell et al., 2011). Hence, family support may moderate the relationship between parental stressors (such COVID-19-related stress and child problem behaviors) and stress related to low SES and might be a key factor to confront children’s psychological issues, especially during isolation times and particularly within low-SES households.

However, still little is known about the interplay of factors that might protect children from the development of physical and emotional health problems during a health emergency in low- compared with high-SES families. To fill this gap, the main aim of the present study was to explore how pandemic-related variables and parental subjective experience of COVID-19-related stress could affect school age children’s self-reported physical and emotional health for 3 months (March, April, and May 2020) of lockdown. Moreover, we assessed whether parental stress is differently linked to children’s physical and emotional health among low- and high-SES families (inclusion in either high or low SES group was based on family income, parental education, and employment status) and whether a supportive family environment worked as a protective factor. Specifically, three research questions (RQs) guided the study:

RQ1: What are the most frequent sources of stress reported by parents because of the pandemic and lockdown, and how do they differ between high- and low-SES households?

RQ2: Are specific sources of parental stress differently linked to the physical and emotional discomfort experienced by children in low- and high-SES families?

RQ3: Is children’s physical and emotional discomfort directly or interactively influenced by SES, parental stress related to COVID-19, and family support?

Based on the recent literature, we expect a moderating protective role of family support, especially among low-SES households and when less distress is experienced by parents. That is, parents who report to experience less stress in relation to the pandemic might be more supportive, and in turn, children should report less discomfort. This should be particularly true among families with low household income, where supportive families (and probably less parental stress is reported) should increase children’s well-being (Crosnoe et al., 2010; McConnell et al., 2011).

## Method

### Participants

The sample included 116 children, 61 boys (53%), with a mean age of 8.70 (*SD* = 1.33; range = 6–11), and their parents. Based on family total income, parental education and rate of parental unemployment, families were grouped into low and high SES. Specifically, 60 (52.2%) were fromfamilies with low household income and 56 (47.8%) from families with medium- to high household income- (See Table 1 for a complete description). It should be noted that according to the National Institute of Statistics (Istat, 2020) in 2020 in Italy a household with four members with less than 20,000 euros per year is considered living below the poverty line. Families with low household income were all either below the poverty line (*n* = 40, 66.6% of the sample) or less than 1 SD above (*n* = 6, 10%). Since participants were recruited thanks to an academic tutoring program (see below) 95% of participants asked to take part in the study agreed to do so and the drop-out rate was zero.

**Table 1:** Descriptive statistics on demographic variables and comparison between low and high SES families

	Low SES *N* = 60	High SES *N* = 56		
	M (SD)	M (SD)	Range	Group Comparison
Child age	8.73 (1.38)	8.67 (1.29)	6–11	*t*(116) = 0.24, *p* = 0.81
Child gender (male)	30 (50%)	32 (56.4%)		χ^2^ (2) = 0.59, *p* = 0.44
Number of family members within the household	5.63 (1.72)	3.21 (0.34)	2–10	*t*(112) = 8.74, *p* = 0.001
Mother education (in years)	9.96 (4.29)	17.45 (3.89)	5–21	*t*(112) = −9.60, *p* = 0.001
Father education (in years)	10.63 (4.23)	16.49 (4.08)	5–21	*t*(112) = −7.01, *p* = 0.001
Family income:				χ^2^ (6) = 65.91, *p* = 0.001
I’d rather not answer	14 (23.3%)	11 (20%)		
<12.000 euro	25 (41.7%)	0		
12.000–19.000 euro	15 (25%)	0		
20.000–29.000 euro	6 (10%)	0		
30.000–49.000 euro	0	24 (41.9%)		
50.000–70.000 euro	0	13 (23.6%)		
>70.000 euro	0	8 (14.5%)		
Employment status				
Both parents unemployed (yes)	11 (18.3%)	0 (0%)		χ^2^ (2) = 8.73, *p* = 0.001
One parent unemployed (yes)	30 (50%)	2 (0.4%)		χ^2^ (2) = 31.79, *p* = 0.0001
Number of rooms in the house	2.76 (1.17)	3.91 (1.25)	1–8	*t*(116) = −4.97, *p* = 0.001
Lack of a family routine	3.48 (0.98)	1.87 (0.92)	1–5	*t*(115) = 3.61, *p* = 0.001
Days in lockdown	42.52 (31.32)	35 (27.50)	0–120	*t*(115) = −1.44, *p* = 0.17
Danger COVID-19	4.3 (0.94)	3.73 (0.70)	1–5	*t*(115) = −4.80, *p* = 0.001
Knowing somebody with COVID-19 (Yes)	3 (5%)	21 (37.5%)		χ^2^ (2) = 16.02, *p* = 0.001
Family support	3.52 (0.86)	3.71 (0.77)	0–5	*t*(115) = 1.27, *p* = 0.21

### Procedure

The present cross-sectional and multi-informant study obtained ethical approval from the university review board (blank for review purpose). Families were recruited through collaboration with schools; more specifically by means of a project of academic tutoring offered during the pandemic by a network of volunteers who supported children and families that either had no access to regular on-line lessons (e.g., no device available in the family) or needed guidance to do so (e.g., parent busy working and unable to support the child with connection). Given that the researchers knew the families through this project during one of the tutoring calls they asked whether they were willing to take part in a study assessing children’s emotional and academic functioning while in lockdown. Parents whom expressed their willingness to participate where given a link containing a written text describing the study followed by the informed consent. Parents that gave their informed consent through the online form where enrolled in the study together with their children. All parents and children were video interviewed between May 11 and 30, 2020. First, caregivers were interviewed. Sociodemographic information was collected with data on COVID-19 exposure and perception. Subsequently, they were asked to report on their main sources of stress because of the pandemic. Once the interview with the parent was over, the child was video-interviewed on their perception of the received family support, COVID-19-related fear or worry, and overall physical and emotional discomfort. Children were invited, when possible, to move to a separate room of the house to guarantee their privacy to answer freely. Once the interviews were over, the parents and children were given some general psychoeducational information on stress management.

### Measures

#### Children’s physical and emotional health

Children were interviewed using the Child Health and Illness Profile-Child Edition (CHIP-CE; Riley et al., 2004). The CHIP-CE is a 45-item questionnaire that can be administered as an interview to a child. It is designed to evaluate the well‐being of children aged 6 through 11 years and examines aspects of health and well‐being that can be influenced by health systems, school systems, and health promotion efforts. The CHIP-CE targets aspects of health-related quality of life that are of special interest to the school-aged group. In the present study, we used the Emotional and Physical Comfort subscales (experience of emotional and physical symptoms, e.g., “In the past 4 weeks, how often did you feel very sad?”; “In the past 4 weeks, how often did you have a bad stomachache?”) and created a general physical and emotional discomfort scale. Ten questions assess the frequency of symptoms in the past 4 weeks using a 5-point scale (0 = never, 4 = always).

In the present study, the scale showed fairly good internal consistency, with a Cronbach’s alpha of 0.79.

#### Family support

The Family Support scale of the CHIP-CE (Riley et al., 2004) was used to assess the family support perceived by the child (e.g., “How many times did your parents listen to your thoughts and worries?”). Five questions assess the support perceived in the past 4 weeks using a 5-point scale (0 = never, 4 = always). The scale showed good internal consistency, with a Cronbach’s alpha of 0.80.

#### COVID-19 and lockdown stress for parents

A questionnaire was developed to assess the amount of stress experienced by the parents of both groups (low- and high-SES) of children. They were asked to report on a 5-point scale (0 = never, 4 = always) how much, during the last 2 weeks, the listed events had been stressful for them. Examples of events were dealing with new family routines (e.g., organization of the day, household, or space), work (or lack of work), social relationships (e.g., children and partner), and online schooling (e.g., management of children’s homework). Stress related to COVID-19 uncertainty (or future plans), its danger, and fear for personal or others’ health was also measured. The internal consistency of the questionnaire was high (Cronbach’s alpha = 0.89).

## Results

### Data Analyses Plan

All data were analyzed in the R environment (R version 4.1.2). First, descriptive analyses were performed on all study variables together with relevant correlations. Subsequently, a series of *t* tests for independent samples were performed to assess whether the sources of stress reported by parents because of the pandemic and lockdown differed between high- and low-SES households. Pearson’s correlations were performed between parental sources of stress related to COVID-19, lockdown, and children’s perceived physical and emotional discomfort. Subsequently, the Fisher’s *z* test of significance between correlation coefficients (Diedenhofen & Musch, 2015) allowed to control for the possibility of an observed difference occurring simply by chance.

Afterward, for the sake of simplicity, all the sources of parental stress experienced as a consequence of the spread of COVID-19 and the lockdown were grouped into two COVID-19 stress indexes: one related to the spread of the virus itself (i.e., stress related to COVID-19 uncertainty and stress related to fear for one’s own or others’ health) and one related to the lockdown (i.e., stress related to dealing with new family routines, work, social relationships, and online schooling). A confirmatory factor analysis was used to assess whether the two-factor model fitted the data well. For each dimension (i.e., stress related to COVID-19 and stress related lockdown), the sum of the items was computed. Last, a linear regression allowed to assess if children’s physical and emotional discomfort was directly or interactively influenced by SES (low vs. high), parental stress related to COVID-19 and lockdown, and children’s perceived family support^1^ (RQ3). Starting from the baseline theoretical full model, we used a model selection approach based on the Akaike information criterion (AIC) to find the most plausible model based on the observed data (American Psychological Association and Akaike, 1987; Wagenmakers & Farrell, 2004). In the tested model, we included all main effects of our study variables (i.e., SES, family support, parental stress related to COVID-19 and lockdown), as well as their two- and three-way interactions. We relied on an exploratory rather than confirmatory model selection approach, based on the assumption that children’s physical and emotional discomfort is a very complex phenomenon that can hardly be captured in a single confirmatory model. Results were interpreted by calculating an evidence ratio comparing the AIC of the best fitting model and the AIC of the baseline model (American Psychological Association and Akaike 1987; Wagenmakers & Farrell, 2004). To explore eventual significant two- or three-way interaction effect, we performed tests of the simple slopes using the jtools package (Long, 2018) in R.

### Preliminarily Analyses

Descriptive statistics and group comparisons between the main demographic characteristics of the low- and high-SES families are reported in Table 1. As can be seen, the two groups were significantly different in terms of mother and father education, income, rate of unemployment (both or single parent unemployed), number of rooms in the house, and lack of a routine during the lockdown. In addition, families with low household income were more scared of COVID-19, even if they knew significantly fewer people who had fallen ill with the virus.

### Parental Stress, Children’s Physical and Emotional Discomfort

Parents were interviewed on the major sources of stress related to both the spread of COVID-19 and the consequent months of lockdown. The most relevant sources of stress were uncertainty related to the spread of COVID-19 (*M* = 3.20, *SD* = 0.89) and fear for their own or others’ health (*M* = 2.49, *SD* = 1.00). Moreover, the lockdown caused significant distress related to having to deal with online schooling (*M* = 2.83, *SD* = 0.94), new family routines (*M* = 2.41, *SD* = 0.77), and work or lack of work (*M* = 2.66, *SD* = 0.80).

Table 2 shows how the sources of stress reported by parents because of the pandemic and lockdown differed between high- and low-SES households. Overall perceived stress related to COVID-19 was higher among families with low household income, except for online schooling and social relationships, which were perceived as more distressing by high-SES families. However, the dissimilarities between the amount of stress perceived by the two groups were significantly different only in relation to COVID-19 uncertainty, *t*(114) = 3.98, *p* = 0.001, fear for one’s own or others’ health, *t*(114) = 3.29, *p* = 0.001, and stress related to online schooling, *t*(114) = 1.23, *p* = 0.028.

**Table 2:** Descriptive statistics of parental sources of stress and relationships with children’s physical and emotional discomfort

	High SES		Low SES		
	Correlations with children’s physical and emotional discomfort	M (SD)	Correlations with children’s physical and emotional discomfort	M (SD)	Range
Children’s physical and emotional discomfort	–	6.35 (1.25)	–	6.61 (1.79)	1–9
COVID danger	0.27*	3.37 (0.71)	0.12	4.41 (0.72)	2–5
Stress related to dealing with new family routines	0.33*	2.34 (0.79)	0.12	2.49 (0.78)	1–5
Stress related to work (or lack of work)	0.15	2.48 (0.66)	0.09	2.74 (0.89)	1–5
Stress related to social relationships	0.31*	2.10 (0.87)	0.28*	1.87 (1.04)	1–5
Stress related to on line schooling	0.38**^a^	2.83 (0.80)	−0.12^a^	1.63 (0.94)	1–5
Stress related to COVID uncertainty	0.41**^b^	2.88 (82)	0.17^b^	3.51 (0.89)	1.5–5
Stress related with COVID-fear for own’s or others’ health	0.37**	2.19 (0.89)	0.29*	2.78 (1.0)	1–5

^a^Correlation coefficients were statistically different with Fisher’s *z* = 2.73, *p* = 0.01.

^b^Correlation coefficients were statistically different with Fisher’s *z* = 1.83, *p* = 0.05.

*p  <  0.05; **p  <  0.01

Table 2 reports the correlations between parental sources of stress related to COVID-19, lockdown, and children’s perceived physical and emotional discomfort. Interestingly, the amount of stress reported by parents in relation to COVID-19 and the subsequent lockdown was positively associated with children’s distress mainly among high-SES families. Fisher’s *z* test revealed the presence of only two statistically different coefficients. As reported in Table 2, among high-SES households, parental stress associated with online schooling was significantly and positively associated with child discomfort, whereas this association was lower and nonsignificant among families with low household income (*z* = 2.73, *p* = 0.01). Similarly, parental stress related to COVID-19 uncertainty was significantly related to children’s discomfort in the families with high but not in the families with low household income (*z* = 1.83, *p* = 0.05).

All the sources of parental stress experienced because of the spread of COVID-19 and the lockdown were grouped into two COVID-19 stress indexes that fitted the data well. Specifically, the first factor was related to the spread of the virus itself (i.e., stress related to COVID-19 uncertainty and stress related to fear for one’s own or others’ health) and the second to the lockdown (i.e., stress related to dealing with new family routines, work, social relationships, and online schooling): T1: *χ*^2^(4) = 1.85, *p* = 0.76, CFI = 1.00, TLI = 1.00, RMSEA = 0, 90% CI [0, 0.09], SRMR = 0.02.

### Influence of Parental Stress, Socioeconomic Status, and Perceived Family Support on Children’s Physical and Emotional Discomfort

To answer the third research question, we performed a linear regression to assess if children’s physical and emotional discomfort was directly or interactively influenced by SES (low vs. high), parental stress related to COVID-19 and lockdown, and children’s perceived family support. In the initial model, we also included child age, gender, fear of COVID-19, and days in lockdown as covariates. Yet these variables were not selected in the final model (and also when included did not change the results), hence for the sake of simplicity they were left out from the beginning.

In the final model, we included all main effects of our study variables (i.e., SES, family support, parental stress related to COVID-19 and lockdown), as well as their two- and three-way interactions. We relied on an exploratory rather than confirmatory model selection approach, based on the assumption that children’s physical and emotional discomfort is a very complex phenomenon that can hardly be captured in a single confirmatory model. The best fitting model (AIC = 77.46; AIC_w_ = 0.41) predicting children’s physical and emotional discomfort is presented in Table 3. The associated evidence ratio showed that this model was 41 times more likely to have generated the observed data than the baseline model (AIC = 86.67). This model explained 19% of the variance of children’s physical and emotional discomfort. The model included the significant main effects of SES and family support. Moreover, one two-way interaction was found between SES and family support.

**Table 3:** Linear regression model predicting children’s physical and emotional discomfort

Predictor	*B (SE)*	*t*	*p*	*η* ^*2*^_*p*_
SES (Low and High)	−17.91 (7.25)*	−2.25	0.03	0.13
Family Support	−4.81 (1.78)*	−2.69	0.023	0.06
Stress Lockdown	−0.005 (0.023)**	−1.557	0.009	0.07
Stress COVID-19	−1.67 (1.56)	−1.071	0.29	0.04
SES x Family Support	5.10 (2.17)	2.34	0.02	0.03
SES x Stress COVID-19	5.46 (2.75)	1.99	0.05	0.03
Family Support x Stress COVID-19	0.58 (0.44)	1.31	0.19	0.01
Family Support x Stress Lockdown	0.86 (0.50)	1.74	0.08	0.02
SES x Family Support x Stress COVID-19	−1.53 (0.75)	−2.03	0.04	0.05
Total R^2^	0.19			
*N*	115			

Note: Baseline category for SES group was families with low household income.

*p < 0.05; **p = < 0.01

**Fig. 1:**
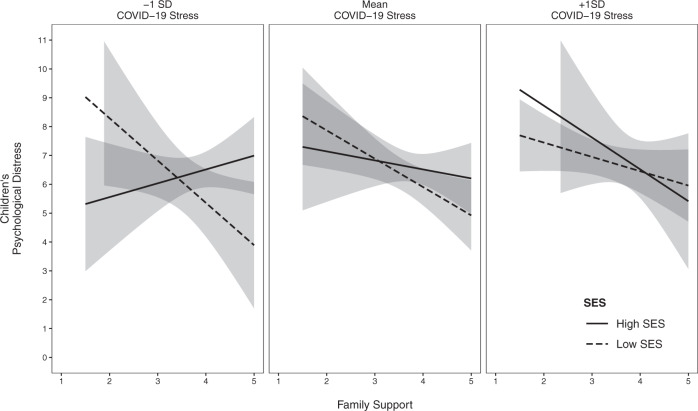
Three-way interaction between SES, family support, parental stress related to COVID-19 predicting children’s physical and emotional discomfort

In addition, a three-way interaction between SES, family support, and stress related to COVID-19 was found (Fig. 1). Simple slope analysis showed that within families with low household income, the slope of family support was significant when parents perceived low (−1 SD; *B* = −1.47, *SE* = 0.68, *t* = −2.15, *p* = 0.03) and average stress in relation to COVID-19 (*B* = −0.98, *SE* = 0.38, *t* = −2.56, *p* = 0.01). Children who perceived higher support from their parents reported less physical and emotional discomfort compared with those who reported lower support. Differently, when the levels of COVID-19 stress were high (+1 SD), children’s psychological complaints were not affected by perceived family support (*B* = −0.50, *SE* = 0.32, *t* = −1.56, *p* = 0.12). Instead, among high-SES families, the slope of family support was not significant for low (*B* = 0.48, *SE* = 0.50, *t* = 0.97, *p* = 0.34), average (*B* = −0.31, *SE* = 0.47, *t* = −0.67, *p* = 0.51), or high (*B* = −1.10, *SE* = 0.88, *t* = −1.25, *p* = 0.21) levels of COVID-19-related stress.

## Discussion

This study investigates the interplay of factors that might affect children’s own perception of their physical and emotional discomfort during a health emergency in low- compared with high-SES families. Furthermore, we assessed how parental sources of stress in response to COVID-19, together with family support, affected children’s psychological adjustment in low- and high-SES households.

Findings revealed that the most frequent sources of stress reported by parents were related to the spread of COVID-19 and the uncertainty associated with it. Although the lockdown also caused significant distress, this was rated as slightly less disturbing. These data seem to be in line with previous studies reporting how uncertainty is a powerful trigger of a stress response and worrisome thinking (Greco & Roger, 2003; Reuman et al., 2015) which is probably due to the novelty of the virus, the presence of asymptomatic patients which do not allow to be constantly aware of who might infect us thus increasing the lack of control, and the lack of scientific knowledge on how to fight it (e.g., at that time no vaccine was available; Slovic, 1987). Indeed, a well-known link exits between intolerance of uncertainty and mental health problems; a relationship that was exacerbated during the pandemic (Jansenn et al., 2020; Raizer et al., 2021). In addition, recent literature has shown how the COVID-19 pandemic and all the required behaviors to contain the spread of the virus caused a chronification of uncertainty about the future, as well as a significant diminished life course agency, especially for parents in more insecure situations (Sanchez-Mira et al., 2021).

Interestingly, on the one hand, perceived stress related to COVID-19 was higher among families with low household income who reported being more worried about the uncertainty and for their own health. On the other hand, high-SES families were more distressed by online schooling and social relationships compared with parents with low household income. One possible explanation for such differences in sources of parental distress in the two groups could be that within families with low household income, parents were less educated and probably did not have all the resources needed to adequately assess the situation (Barg, 2019; Bradley et al., 1989; Miller et al., 2014). This could have led them to rely more on heuristic judgments and to use their feelings as a cue to assess the threat of the virus (Loewenstein et al., 2001; Martel et al., 2020; Slovic & Peters, 2006). In other words, stressed individuals who lack control over a problem (such as parents that are unable to assess the risk of the COVID-19) are prone to make predictions based on their emotional perceptions of the threat (Martel et al., 2020; Tompkins et al., 2018).

Compared with parents with low household income, parents with higher household income were more distressed by the changes in the routine related to the need to help their children with online schooling. The rationale here might be that within high SES households, parents tend to be more involved in their children’s learning processes and opportunities (Baquedano-Lopez, et al., 2013). Indeed, literature supports the idea that parental involvement in their children’s education is not equally distributed with socio economic class and status (Baquedano-Lopez, et al., 2013). For example, immigrant and low household income parents are generally less involved in both school activities and helping children with at home learning (Antony-Newman, 2019). Previous studies have demonstrated that parents who live in lower SES households, characterized by chronic poverty and poorer parental education, often spend less time on their children’s education (Del Bono et al., 2016; Guryan et al., 2008) and provide less stimulating learning materials and experiences (Bradley et al., 1989; Miller et al., 2014). High-SES parents might also feel more knowledgeable on how to support their children’s learning compared with immigrant families with low household income, because they had more learning opportunities themselves and they know how to deal with schools and teachers (Harris & Goodall, 2008; Minke et al., 2014). Families with low household income might prefer to totally rely on teachers as the best possible solution for their children’s education, because they experience a lot of difficulties in assisting their children’s schooling, due to language limitations, less time, financial constraints, and poor prior negative experiences with schools (e.g., Barg, 2019; Hill & Torres, 2010; Ishimaru et al., 2016). As a consequence, high-SES parents were more engaged in schooling whereas parents with low household income were less involved in their children’s on-line education, resulting in less stress regarding children’s schooling compared to other more pertinent sources of stress (Addi-Raccah & Seeberger Tamir, 2022). In addition, families with high household income might have been more impacted from school closure and the absence of after school activities, that are usually a great support to regular daily activities (Wang et al., 2020).

The different sources of parental stress were also differently linked to the physical and emotional discomfort experienced by children. Interestingly, it was mainly within high-SES environments that parental reported stress was linked with children’s physical and emotional discomfort. That is, significant correlations between parental stress and child psychological problems were present more in high-SES families. However, these associations statistically differed in the two groups only mildly. Parental stress about homeschooling was linked with child discomfort only among high-SES families. This might be explained by the greater involvement and investment that high-SES parents dedicate to their children’s schooling, which regularly promotes achievement, motivation, and success (Froiland, 2020; Li et al., 2020), but that might become distressing when dealing with the online schooling. These parents might expect their children to have high performance and might feel responsible for unexpected difficulties or overwhelmed by the amount of work needed to supervise the schoolwork. Such distress could have placed great pressure on children, who indeed reported higher discomfort. In the opposite direction, children experiencing more distress could have been more difficult to deal with during online schooling, which could have increased parental hardship. This association was not present among families with low household income, where other sources of stress might be more relevant in the family routine.

Another significant difference was found in the association between parental stress related to uncertainty due to COVID-19 and child emotional and physical discomfort. Once again, this association was present only within high-SES households. The rationale here could be that children belonging to families with low household income are used to deal with numerous sources of distress, including being indirectly exposed to parental stress. A possible explanation might be that those living in a low-SES contexts may put greater effort in trying to accept stressors and to adjust oneself to deal with stressful situations through different strategies. According to the “Shift-and-persist” model (Chen & Miller, 2012), when placed in a stressful environment, people from low-SES background may adopt several strategies to deal with the situation itself, such as reappraisals (what they call shifting), enduring adversity with strength, or maintaining optimism in the face of adversity (what they call persisting). Shift-and-persist strategies have been linked not only to better physical health but also to better mental one (Nakashima & Lee, 2016; Lee & Nakashima, 2019). This might have made them more capable of handling difficult situations involving COVID-19-related stress. Overall, the threats related to COVID-19 include an increase of demands on the parent–child dyad, also associated with a potential reduction in parental capacity due to the increased stress levels (Prime et al., 2020). This seems to be related with children’s psychological functioning, especially among high-SES families, where uncertainty related to COVID-19 and life uncertainty, was something that they were not reused to handle. Hence, more resources were needed to adjust to such change and uncertainty. More information is needed here to clarify these findings; however, the existing literature shows that children’s adjustment is largely contingent on the general climate within the family (Browne et al., 2015). Supportive environments characterized by cohesion and positive affective climates is a strong protective factor; even in response to the COVID-19 some preliminary data have shown how a positive dialogue within families is a significant protective factor in preventing youths’ mental health (Tang et al., 2021).

However, children can also be resilient when facing cumulative sources of stress and trauma (Masten & Narayan, 2012). That is, while more disadvantaged families that already carry the cumulative consequences of chronic sources of stress may be more vulnerable on the long term, they could be more capable to quickly respond to the new challenges caused by the pandemic adopting the same strategies previously used to handle the threats in their environment.

Finally, we investigated whether physical and emotional discomfort was directly or interactively influenced by low SES, parental stress related to COVID-19, and family support. The findings are in line with the model proposed by Prime et al. (2020), which explains how COVID-19 might impact child adjustment in a cascading fashion. Following this model, social disruptions from the pandemic (e.g., job issues, insecurity, and confinement) affect caregiver well-being (e.g., parenting stress and psychological distress), which in turn influences children’s adjustment. Importantly, this model also clearly states the relevance of preexisting family vulnerabilities, such as low SES, that directly and indirectly affect the entire process. The model proposed by Prime et al. (2020) underlines the importance of several moderating factors that will be at play, placing some families and children at heightened risk for poor outcomes and others in a position to thrive and adapt to the situation.

In line with this, we found a significant three-way interaction between SES, COVID-19-related stress, and family support. Specifically, when the COVID-19-related stress perceived by parents was low or average, greater family support significantly improved children’s physical and emotional health, but only in low-SES environments. These data seem to indicate that perceived family support is particularly important for child psychological well-being among families with low household income. Previous literature repeatedly reports how family relationships and support are implicated during stress and major life events (Doom & Cicchetti, 2018; Masten, 2016; Masten & Narayan, 2012). In addition, families with preexisting strengths are often those that can still be supportive in times of major life-threatening events; such strengths represent an important protective factor for child adjustment and thriving (Doom & Cicchetti, 2018; Masten, 2016; Masten & Narayan, 2012). The present data may suggest that families with low household income might be capable of offering sufficient support to their children to help them better deal with the psychological strain of the pandemic. However, this effect is only significant when parental stress related to COVID-19 is low to moderate. That is, when perceived stress is high, it probably represents an unbearable threat that undermines all other processes. In other words, it could be that for families with low household income, the initial level of stress is already quite high. Hence, family support can help overcome the baseline stress plus an additional low amount of stress related to COVID-19, but when the emergency induces overly high stress responses, in addition to the baseline, family support fails to protect the child.

It is worth mentioning that when parents perceived high stress related to COVID-19, in both high- and low-SES environments, the degree of family support perceived by children did not significantly protect them from physical and emotional discomfort. When the sources of stress related to uncertainty and fear become too high, not even family support is enough to protect children from physical and emotional discomfort. This could be characteristic of the current health emergency and the multiple secondary stressors associated with it. Considering a family system model (Fiese et al., 2019), we might propose that the interconnections between family members are disrupted by the COVID-19 uncertainty and fear, as well as by all secondary stressors associated with it (e.g., being confined at home, limited space, marital conflict). Such troubles within the family system could undermine the “efficacy” of the usually protective role of family support on children’s adjustment.

The present study is not free from limitations. First, the sample size was small, and consequently, the power of the analyses is limited (0.96). Thus, the study needs to be replicated to make the findings more generalizable. However, this study was just a preliminary investigation of a subject that needs to be further explored. In addition, the small sample size did not allow us to control for several possibly relevant covariates related to sociodemographic information, parenting style, and parenting functioning. In addition, we lacked a measure of parental mental health, which might have affected children’s well-being and adjustment. Furthermore, due to the cross-sectional nature of the study, it is not possible to make a causal inference based on our results. Finally, it would have been interesting to assess several individual characteristics of both children and parents that could potentially moderate the outcome (e.g., emotion regulation abilities).

Despite these caveats, this was the first study to directly ask children how they psychologically adjusted to the pandemic and to assess the effects of parental stress and SES on children’s response to COVID-19.

Overall, there is a major concern about the acute and long-lasting impact of COVID-19 on children’s well-being. The present data, in line with recent findings on youth’s mental health (Loades et al., 2020; Racine et al., 2020), show that children themselves are indeed feeling emotionally and physically distressed by the health emergency. The pandemic represents not only a public health and economic global crisis, but also a challenge to the well-being of children and families. Here, we show some evidence of the negative cascade that flows from parental stress due to the pandemic and related lockdown to children’s physical and emotional discomfort; we also show how low-SES households can be more at risk than their high-SES counterparts. Interestingly, however, we found some evidence that poorer environments might have somehow prepared families and children to better deal with major crises, including the COVID-19 emergency. Importantly, especially within these families, family support is an important protective factor when facing the effects of pandemic-related stress.

This could have important implications for social policy and mental health practitioners, who should be aware that the cumulative negative effects of COVID-19 can be interrupted with significant effort in promoting family well-being and support. That is, especially in poorer environments, children might significantly benefit from the buffering role of a supportive family against the effects of parental COVID-19-related stress.

## Data Availability

The data that support the findings of this study are available on request from the corresponding author, SS. The data are not publicly available due to their containing information that could compromise the privacy of research participants.
